# Extraparenchymal neurocysticercosis in the United States: a case report

**DOI:** 10.1186/1752-1947-5-359

**Published:** 2011-08-09

**Authors:** Theodoros Kelesidis, Sotirios Tsiodras

**Affiliations:** 1 Department of Medicine, Division of Infectious Diseases, David Geffen School of Medicine at UCLA, Los Angeles, California, USA; 2 Fourth Department of Internal Medicine, Attikon University Hospital, University of Athens Medical School, Athens, Greece

## Abstract

**Introduction:**

Neurocysticercosis is endemic in the developing world, but is becoming more common in the United States due to immigration.

**Case presentation:**

A 26-year-old Caucasian man presented with headache, nausea and vomiting and was found to have hydrocephalus and meningitis. Brain imaging and immunological studies were suggestive of neurocysticercosis. Endoscopic removal of the cyst resulted in resolution of symptoms. This case represents a combination of two rare presentations of extraparenchymal neurocysticercosis; intraventricular neurocysticercosis and subarachnoid neurocysticercosis.

**Conclusion:**

Although neurocysticercosis is pleomorphic in its presentation, extraparenchymal neurocysticercosis may be challenging to diagnose and treat. Clinicians should be aware of this condition given increasing incidence in the United States.

## Introduction

Neurocysticercosis (NCC) is the most common disease causing cystic lesions in the central nervous system, especially in developing and tropical countries [1]. However, extraparenchymal NCC may be challenging to diagnose and treat. Increasing immigration from endemic areas will lead to an increasing frequency of extraparenchymal NCC in the United States. Clinicians and neuroradiologists in the United States are often unaware of the radiographic patterns of extraparenchymal NCC and the potentially poor prognosis if not correctly diagnosed and managed. Here, we describe a case of extraparenchymal NCC as a cause of chronic meningitis and hydrochephalus in a patient and we discuss challenges in the diagnosis and management of these cases.

## Case presentation

**Figure 1:**
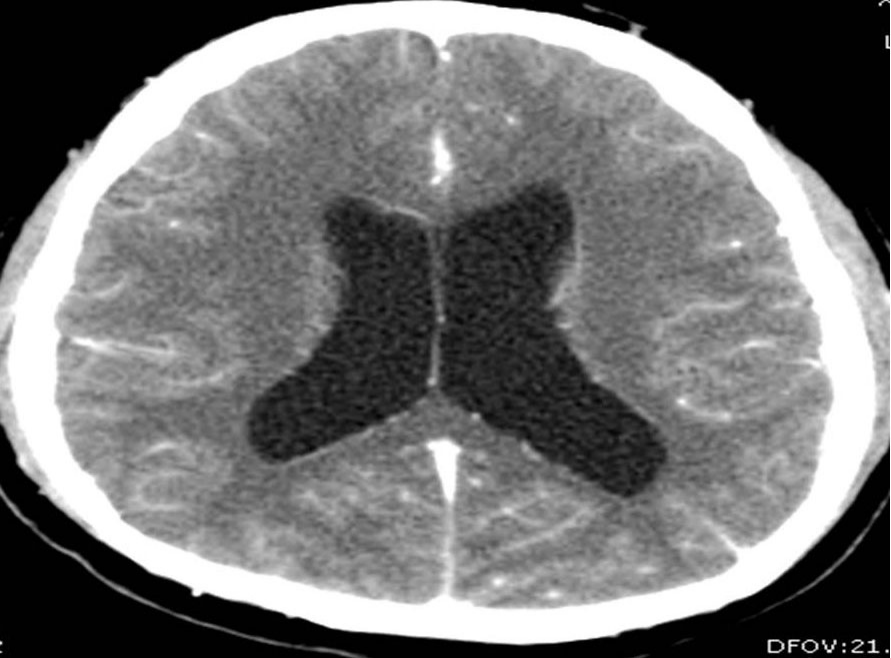
**CT of his head (transverse view) showing presence of hydrocephalus and dilation of ventricles**.

**Figure 2:**
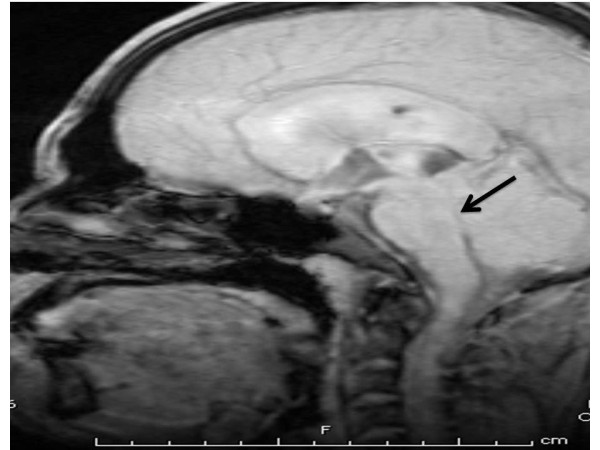
**Midsagittal T1-weighted MRI of his head showing hydrocephalus with stenosis of aqueduct (*arrow*)**.

**Figure 3:**
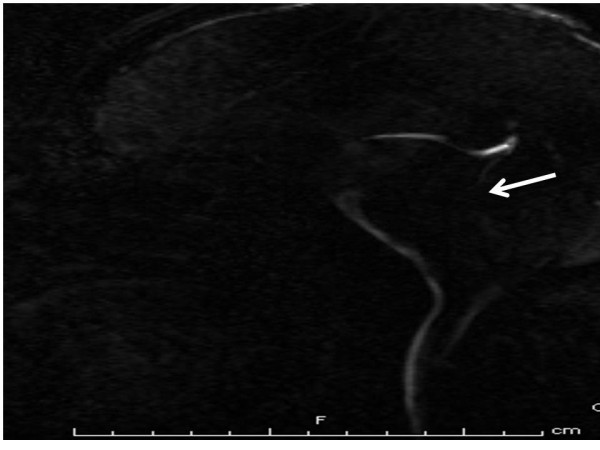
**Cine flow MRI: lack of cerebral aqueduct flow void (arrow) but no obvious cyst**.

**Figure 4:**
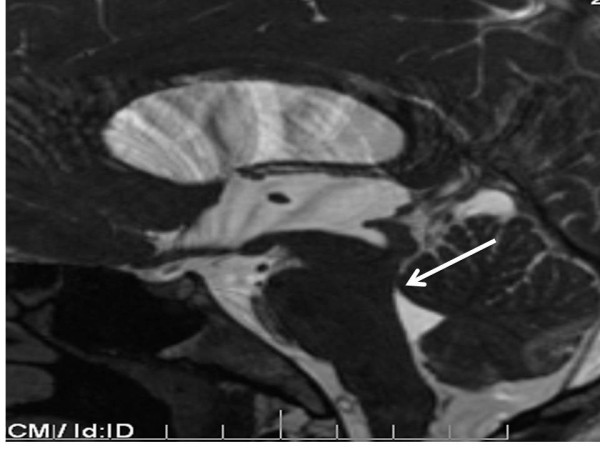
**Midsagittal T2-weighted MRI of his head showing hydrocephalus with lack of cerebral aqueduct patency (*arrow*)**.

**Figure 5:**
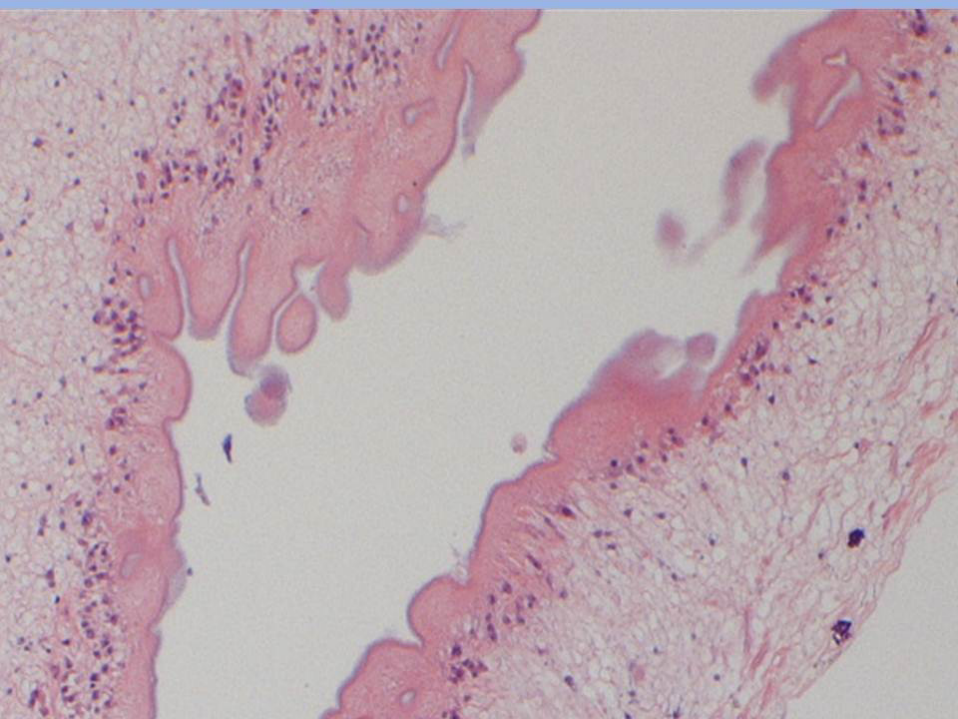
**Pathology specimen**. Wavy, somewhat refractile cuticle with presence of loose, reticular tissue.

A 26-year-old Caucasian man with history of diabetes mellitus presented to our emergency department complaining of diffuse headache that had developed two months prior to presentation. He also reported nausea and vomiting that became progressively worse. He denied fever, chills, photophobia or any other symptoms. He was born in Mexico and moved to California when he was 18 years old. A physical examination revealed a temperature of 37°C and mild neck stiffness but an examination including funduscopy and ophthalmologic examination was otherwise normal. Initial laboratory findings indicated a white blood cell count of 6500/mm^3 ^with 75% polymorphonuclear leukocytes, 15% lymphocytes and 8% monocytes. His serum glucose was 90 mg/dL. The remainder of the laboratory data was normal. An initial computed tomography (CT) scan of his head showed hydrocephalus (Figure 1). A lumbar puncture was performed and cerebrospinal fluid (CSF) analysis revealed an opening pressure of 400 mmHg, with 330 white blood cells/mm^3 ^with 14% neutrophils, 45% lymphocytes, 38% monocytes, 3% eosinophils, 3 red blood cells/mm^3^, a protein level of 84 mg/100 mL and a glucose level of 43 mg/100 mL. A Gram stain and India ink stain of CSF were negative and final bacterial, viral, fungal and mycobacterial cultures were negative. Our patient was initially started on antimycobacterial therapy for empiric treatment of possible tuberculous meningitis. Magnetic resonance imaging (MRI) of his brain revealed an absence of meningeal enhancement, dilated lateral and third ventricles, with aqueduct stenosis and a possible small cystic area in or adjacent to the inferior aqueduct (Figures 2, 3, 4). An enzyme-linked immunosorbent assay of the CSF was positive for immunoglobulin G (IgG) cysticercosis antibody, with 1.31 optical density units (OD) (positive result > 0.50 OD). His serum IgG cysticercosis antibody was positive with 4.42 OD. The serological diagnosis was confirmed by enzyme-linked immunoelectrotransfer blot assay performed for both serum and CSF. On further history, our patient mentioned that he used to eat undercooked meat when he was in Mexico. A diagnosis of extraparenchymal NCC was made. Our patient underwent endoscopic removal of the cyst and three weeks of therapy with albendazole (15 mg/kg/day) and corticosteroids was initiated. Postoperatively, our patient had a full range of extraocular movements, no periaqueductal injury and complete resolution of his presenting symptoms. Pathology findings confirmed the diagnosis of NCC (Figure 5).

## Discussion

NCC is the most common parasitic infestation of the central nervous system worldwide but is also of emerging importance in the United States, especially in areas with high volumes of immigration from endemic regions of Latin America [2]. A recent study reported an overall frequency of subarachnoid cysts in 2%, ventricular cysts in 6%, and hydrocephalus in 16% of NCC cases [2]. Intraventricular NCC, the presence of *Taenia solium *cysts in the cerebral ventricular system, occurs in 7-30% of patients with NCC [3]. According to recent studies, extraparenchymal NCC is probably more frequent than previously thought [3]. We have recently reviewed the pathogenesis, diagnosis, clinical manifestations and treatment of extraparenchymal NCC in the United States [4].

In our case extraparenchymal NCC occurred in an adult male Hispanic immigrant who presented with subacute intracranial hypertension from hydrocephalus and chronic meningitis, characterized by a mild-to-moderate CSF lymphocytic pleocytosis, mild-to-moderate increase in protein, and minimal meningeal signs on exam. Intracranial hypertension is a common manifestation of extraparenchymal NCC and in our case was caused by the direct obstruction of the aqueduct by a cyst and possible blockage of CSF pathways within the subarachnoid space. This blockage may have been due to the inflammatory reaction at the tentorium opening.

Extraparenchymal NCC is associated with a local inflammatory response with high protein concentration and cell counts in the CSF [5]. Clinical manifestations and CSF findings are similar to the more common tuberculous and fungal meningitis [5,6], since the CSF findings consist of pleocytosis (usually lymphocytic but frequently polymorphonuclear), reduced glucose and elevated protein [5]. In one series of cysticercal meningoencephalitis, confusion with tubercular meningitis was present in 61.5% cases [5]. An important differentiating feature is the presence of eosinophils (above 5%) in the CSF, which is usually seen only in the initial phases of the illness but occurs in only 15% of patients [3]. The presence of chronic meningitis, hydrocephalus, and absence of eosinophils in the CSF of a patient coming from an endemic area for tuberculosis suggested the diagnosis of tuberculous meningitis, and our patient was started on empiric treatment for tuberculosis until a diagnosis of neurocysticercosis was made. It is often common practice to attribute chronic meningitis and hydrocephalus to tubercular meningitis in the presence of appropriate epidemiologic history and treat empirically by shunting and antitubercular therapy. Thus, an astute clinical acumen is required to make the diagnosis of cysticercal meningitis.

Neuroimaging findings of extraparenchymal cysticerci are subtle and are usually not seen by CT. The most common CT finding in subarachnoid NCC is hydrocephalus [7]. In our case, there was hydrocephalus and non-specific cerebral aqueduct stenosis but no obvious imaging evidence of a parasite in the parenchymal or extraparenchymal space. The cyst was located in the aqueduct but was very subtle and was initially missed by the radiologist. Because the cyst membrane is thin and the fluid is isodense with the cerebrospinal fluid, uninflamed extraparenchymal cysticerci are usually not visible on CT scanning and may only reveal subtle, indirect findings on MRI [7].

MRI has good sensitivity for detecting intraventricular cysticercosis and although it did not initially clear the diagnostic dilemma in this case, contrast MRI was helpful in clarifying the morphology of the lesion. However, only the presence of cystic lesions, demonstrating the scolex of the larval stage of *T. solium*, can be considered pathognomonic of NCC and an absolute criterion for a definitive diagnosis of the disease [1,8,9]. Thus, previous studies have shown that optimal MRI protocols should include axial fluid-attenuated inversion recovery imaging to obtain maximal rates of scolex detection [8]. In our case the exact etiology of the cystic lesion was confirmed on pathological examination of the specimen.

The diagnosis in our case was made based on the presence of a lesion highly suggestive of NCC on neuroimaging study, positive serum immunoassay for the detection of anticysticercal antibodies, positive CSF immunoassay for detection of anticysticercal antibodies and epidemiologic criteria including an individual coming from an area where cysticercosis is endemic [1]. However, extensive and comprehensive revision of the diagnostic criteria of NCC, especially of extraparenchymal NCC, is mandatory according to many recent publications [5,7,10].

There is still no consensus regarding optimal treatment strategies in patients with intraventricular NCC [3,6,10]. Various therapeutic modalities include antihelminthic medication, microneurosurgical removal, ventriculoperitoneal shunting, and endoscopic management [5]. Medical therapy alone is not favored because of the limited efficacy in such cases, and a risk of developing acute hydrocephalus during the clinical treatment period because of the mobile nature of the intraventricular cyst [3,11]. Thus, surgical evaluation is necessary prior to medical treatment [5] and in patients presenting with acute hydrocephalus, surgery is the only option [3,11]. Endoscopic approaches for intraventricular NCC have been described in recent years and often allow for cyst removal and hydrocephalus treatment, freeing the patient from shunt procedures [12].

Although subarachnoid cysts were not identified, our patient's clinical presentation and CSF analysis was consistent with cysticercal meningitis and subarachnoid cysticercosis. Thus, our case represents a combination of two rare presentations of extraparenchymal NCC; intraventricular NCC and subarachnoid NCC. There was a consensus among treating neurologists, neurosurgeons and infectious disease consultants to use albendazole at a dose of 15 mg/kg/day for a minimum of 21 days as the antiparasitic treatment. Our patient also received steroids for three weeks and the doses and duration used were equal or bigger than the known standard doses to treat parenchymal disease [6]. However, with controversy in the literature over the optimal management of this condition and without further evidence-based guidelines to help management of extraparenchymal NCC, the decision about the total dose and duration of antiparasitic and steroid therapy was at the discretion of the treating neurologist.

## Conclusion

In summary, the case presented here is an important reminder that intraventricular NCC should be considered in the differential diagnosis of obstructive hydrocephalus with the radiographic appearance of a cystic lesion in the third, aqueduct, or fourth ventricle. Extraparenchymal NCC may be a more common form of NCC in the United States than previously thought. Because clinicians in the US outside the southwest US are often unfamiliar with NCC as a cause of chronic meningitis, chronic ventriculitis, or hydrocephalus without obvious cysts, the diagnosis of extraparenchymal NCC often depends on the correct interpretation of neuroimaging which may miss the diagnosis. Thus, meningeal and intraventricular NCC should always be considered by clinicians and radiologists in the differential diagnosis of chronic meningitis and hydrocephalus, particularly in patients from Latin America.

## Consent

Written informed consent was obtained from the patient for publication of this case report and any accompanying images. A copy of the written consent is available for review by the Editor-in-Chief of this journal.

## Competing interests

The authors declare that they have no competing interests.

## Authors' contributions

TK analyzed and interpreted the patient data and was a major contributor in writing the manuscript. ST analyzed the patient data and contributed in writing the manuscript. All authors read and approved the final manuscript.

## References

[B1] del Brutto OH Neurocysticercosis Semin Neurol 2005 25 3 243 251 10.1055/s-2005-917661 16170737

[B2] Wallin MT Kurtzke JF Neurocysticercosis in the United States: review of an important emerging infection Neurology 2004 63 9 1559 1564 15534236 10.1212/01.wnl.0000142979.98182.ff

[B3] Figueroa JJ Davis LE Magalhaes A Extraparenchymal neurocysticercosis in Albuquerque, New Mexico J Neuroimaging 2011 21 1 38 43 10.1111/j.1552-6569.2009.00452.x 20002970

[B4] Kelesidis T Tsiodras S Extraparenchymal neurocysticercosis in the United States Am J Med Sci 2011 in press 10.1097/MAJ.0b013e31823e6565 PMC8053313 22222333

[B5] Cardenas G Jung H Rios C Fleury A Soto-Hernandez JL Severe cysticercal meningitis: clinical and imaging characteristics Am J Trop Med Hyg 2010 82 1 121 125 10.4269/ajtmh.2010.09-0347 20065006 PMC2803520

[B6] Proano JV Torres-Corzo J Rodriguez-Della VR Guizar-Sahagun G Rangel-Castilla L Intraventricular and subarachnoid basal cisterns neurocysticercosis: a comparative study between traditional treatment versus neuroendoscopic surgery Childs Nerv Syst 2009 25 11 1467 1475 10.1007/s00381-009-0933-4 19557421

[B7] Garcia HH Evans CA Nash TE Takayanagui OM White AC Jr Botero D Rajshekhar V Tsang VC Schantz PM Allan JC Flisser A Correa D Sarti E Friedland JS Martinez SM Gonzalez AE Gilman RH Del Brutto OH Current consensus guidelines for treatment of neurocysticercosis Clin Microbiol Rev 2002 15 4 747 756 10.1128/CMR.15.4.747-756.2002 12364377 PMC126865

[B8] Lucato LT Guedes MS Sato JR Bacheschi LA Machado LR Leite CC The role of conventional MR imaging sequences in the evaluation of neurocysticercosis: impact on characterization of the scolex and lesion burden AJNR Am J Neuroradiol 2007 28 8 1501 1504 10.3174/ajnr.A0623 17846200 PMC8134382

[B9] Castillo M Imaging of neurocysticercosis Semin Roentgenol 2004 39 4 465 473 10.1016/j.ro.2004.06.007 15526530

[B10] Kalra V Mishra D Suri A Seth R Garg A Intraventricular neurocysticercosis Indian J Pediatr 2009 76 4 420 423 10.1007/s12098-009-0023-3 19205637

[B11] Goel RK Ahmad FU Vellimana AK Suri A Chandra PS Kumar R Sharma BS Mahapatra AK Endoscopic management of intraventricular neurocysticercosis J Clin Neurosci 2008 15 10 1096 1101 10.1016/j.jocn.2007.10.004 18653345

[B12] Bergsneider M Endoscopic removal of cysticercal cysts within the fourth ventricle. Technical note J Neurosurg 1999 91 2 340 345 10.3171/jns.1999.91.2.0340 10433327

